# Enzalutamide versus Abiraterone Plus Prednisolone for Nonmetastatic Castration-Resistant Prostate Cancer: A Sub-Analysis from the ENABLE Study for PCa

**DOI:** 10.3390/cancers16030508

**Published:** 2024-01-24

**Authors:** Koji Mita, Kouji Izumi, Akihiro Goriki, Ryo Tasaka, Tomoya Hatayama, Takashi Shima, Yuki Kato, Manabu Kamiyama, Shogo Inoue, Nobumichi Tanaka, Seiji Hoshi, Takehiko Okamura, Yuko Yoshio, Hideki Enokida, Ippei Chikazawa, Noriyasu Kawai, Kohei Hashimoto, Takashi Fukagai, Kazuyoshi Shigehara, Shizuko Takahara, Yoshifumi Kadono, Atsushi Mizokami

**Affiliations:** 1 Department of Urology, Hiroshima City North Medical Center Asa Citizens Hospital, 1-2-1 Kameyama-Minami, Asakita-ku, Hiroshima 731-0293, Japan; 2 Department of Integrative Cancer Therapy and Urology, Kanazawa University Graduate School of Medical Science, 13-1 Takaramachi, Kanazawa 920-8641, Japan; 3 Department of Urology, Hiroshima University Graduate School of Biomedical Sciences, 1-2-3 Kasumi, Minami-ku, Hiroshima 734-8551, Japan; 4 Department of Urology, Toyama Prefectural Central Hospital, 2-2-78 Nishinagae, Toyama 930-8550, Japan; 5 Department of Urology, Fukui-Ken Saiseikai Hospital, 7-1 Wadanakacho-Funabashi, Fukui 918-8503, Japan; 6 Department of Urology, University of Yamanashi, 1110 Shimokato, Chuo 409-3898, Japan; 7 Department of Urology, Shobara Red Cross Hospital, 2-7-10 Nishihonmachi, Shobara 727-0013, Japan; 8 Department of Urology, Nara Medical University, 840 Shijocho, Kashihara 634-8521, Japan; 9 Department of Urology, Fukushima Medical University, 1 Hikarigaoka, Fukushima 960-1295, Japan; 10 Department of Urology, Anjo Kosei Hospital, 28 Anjocho-Higashihirokute, Anjo 446-8602, Japan; 11 Nephro-Urologic Surgery and Andrology, Mie University Graduate School of Medicine, 2-174 Edobashi, Tsu 514-8507, Japan; 12 Department of Urology, Graduate School of Medical and Dental Sciences, Kagoshima University, 8-35-1 Sakuragaoka, Kagoshima 890-8520, Japan; 13 Department of Urology, Kanazawa Medical University, 1-1 Uchinadamachi-Daigaku, Kahoku 920-0293, Japan; 14 Department of Nephro-urology, Nagoya City University Graduate School of Medical Sciences, 1 Kawasumi, Mizuho-cho, Mizuho-ku, Nagoya 467-8602, Japan; 15 Department of Urology, School of Medicine, Sapporo Medical University, 16-291 Minami-1-Jo-Nishi, Sapporo 060-8543, Japan; 16 Department of Urology, Showa University School of Medicine, 1-5-8 Hatanodai, Shinagawa-ku, Tokyo 142-8666, Japan; 17 Department of Urology, Ishikawa Prefectural Central Hospital, 2-1 Kuratsukihigashi, Kanazawa 920-8530, Japan; 18 Innovative Clinical Research Center, Kanazawa University, 13-1 Takaramachi, Kanazawa 920-8641, Japan; 19 Medical Research Support Center, University of Fukui Hospital, 23-3 Shimoaizuki, Matsuoka Eiheiji-cho Yoshida-gun, Fukui 910-1193, Japan

**Keywords:** abiraterone, enzalutamide, castration-resistant, nonmetastatic

## Abstract

**Simple Summary:**

The efficacy of abiraterone plus prednisolone (ABI) against nonmetastatic castration-resistant prostate cancer (CRPC) remains unclear. To evaluate enzalutamide and ABI as the first-line treatment for CRPC, we conducted the randomized controlled trial including both metastatic and nonmetastatic CRPC. As a sub-analysis, we focused on nonmetastatic CRPC in this study. ABI and enzalutamide had similar efficacy and safety profiles in patients with nonmetastatic CRPC.

**Abstract:**

Enzalutamide (ENZ) and abiraterone plus prednisolone (ABI) can improve the survival of patients with castration-resistant prostate cancer (CRPC). However, the agent that is more effective against nonmetastatic CRPC remains unclear. To evaluate the agent that can be used as the first-line treatment for CRPC, an investigator-initiated, multicenter, randomized controlled trial (ENABLE Study for PCa) including both metastatic and nonmetastatic CRPC was conducted in Japan. The prostate-specific antigen (PSA) response rate, overall survival, some essential survival endpoints, and safety of patients with nonmetastatic CRPC were also analyzed. In this subanalysis, 15 and 26 patients in the ENZ and ABI arms, respectively, presented with nonmetastatic CRPC. There was no significant difference in terms of the PSA response rate between the ENZ and ABI arms (80% and 64%, respectively; *p* = 0.3048). The overall survival did not significantly differ between the two arms (HR: 0.68; 95% CI: 0.22–2.14, *p* = 0.5260). No significant differences were observed in terms of radiographic progression-free survival and cancer-specific survival between the ENZ and ABI arms (HR: 0.81; 95% CI: 0.35–1.84; *p* = 0.6056 and HR: 0.72; 95% CI: 0.19–2.73; *p* = 0.6443, respectively). Only four and six patients in the ENZ and ABI arms, respectively, had ≥grade 3 adverse events. ABI and ENZ had similar efficacy and safety profiles in patients with nonmetastatic CRPC.

## 1. Introduction

Prostate cancer is the second most common malignancy and the fifth main cause of death in men worldwide [1]. The number of patients with prostate cancer is gradually increasing in Japan, and prostate cancer is currently the most common malignancy in Japanese men [2]. The standard treatment for metastatic prostate cancer is androgen-deprivation therapy (ADT) with an antiandrogen since the progression of prostate cancer usually depends on androgen receptor signaling [3,4]. However, prostate cancer generally progresses to castration-resistant prostate cancer (CRPC), which is unresponsive to ADT and androgen receptor signaling-targeted agents (ARSTs) after a few years of ADT [5]. ARSTs such as enzalutamide (ENZ) and abiraterone plus prednisolone (ABI) can improve overall survival (OS) and radiographic progression-free survival (rPFS) in CRPC patients with metastasis compared to placebo control both before and after docetaxel treatment [6,7,8,9]. ENZ binds to the ligand-binding domain of the androgen receptor strongly and can hinder its translocation of androgen receptor into the cell nucleus [6]. Abiraterone can inhibit CYP17A1, an important enzyme in androgen synthesis, resulting in the depletion of dihydrotestosterone in cancer cells as well as the whole body [10]. Generally, ARSTs solely targeting androgen receptor signaling are regarded as less toxic agents than docetaxel, which affects all cells in the body and induces more severe neutropenia, especially in the Asian population compared to other ethnicities [11]. Therefore, ENZ and ABI are frequently administered as standard first-line treatments for metastatic CRPC in Japan. Nevertheless, no prospective randomized controlled trials investigating the superiority of ENZ and ABI as not sequential use but a single agent in metastatic and nonmetastatic CRPC have been conducted so far. Therefore, we performed a head-to-head investigator-initiated, multicenter, randomized controlled trial (The ENABLE Study for PCa) comparing ENZ and ABI as first-line endocrine therapies before chemotherapy in Japanese patients with CRPC, regardless of metastatic status [12]. The results showed that ENZ did not have any survival benefits compared with ABI. However, it had a better prostate-specific antigen (PSA) response rate and a low severe adverse event (AE) rate in patients with CRPC for the first time. These data suggest that the antecedent use of ENZ to ABI can have possible clinical benefits in populations with CRPC. However, for nonmetastatic CRPC, ENZ is associated with improvements in metastasis-free survival and OS. Nevertheless, there is no evidence of the survival benefit of ABI [13]. We only analyzed the time to PSA progression in nonmetastatic CRPC, and there was no difference between the ENZ and ABI arms in this primary paper. However, the effects of ABI on nonmetastatic CRPC should be evaluated. Furthermore, the PSA response rate (≥50% decline from baseline), OS, some essential survival endpoints, and AEs of patients with nonmetastatic CRPC were analyzed.

## 2. Materials and Methods

### 2.1. Study Design

The ENABLE Study for PCa is a multicenter, investigator-initiated, randomized controlled trial in Japan that compared the use of ENZ and ABI before chemotherapy in patients with CRPC. Data on patient eligibility and treatment were described in detail in the primary paper. Briefly, patients in the treatment arm (1:1) were randomly assigned to receive ENZ 160 mg/day (four 40 mg tablets once a day) or ABI 1000 mg/day (four 250 mg tablets once a day) and 5 mg prednisolone twice a day through the data center at the Innovative Clinical Research Center of Kanazawa University (iCREK). We focused on patients with nonmetastatic CRPC from all patients included in the ENABLE Study for PCa. These patients were analyzed in the current study.

This study was conducted in accordance with the Ethical Guidelines for Medical and Health Research Involving Human Subjects and the 1975 Declaration of Helsinki (revised in 2013). All treatments and examinations for prostate cancer were performed after the patients provided written informed consent before registration. The current study was first approved by the Medical Ethics Committee of Kanazawa University, Kanazawa, Japan (reference number: 2014-031) and then by the institutional ethics committees of the other 15 participating hospitals. This trial was also registered with the University Hospital Medical Information Network (UMIN) Center (identifier UMIN000015529) on 1 November 2014.

### 2.2. Patient Inclusion and Exclusion Criteria

Patient inclusion criteria were (1) pathologically or cytologically confirmed prostate cancer with castration resistance defined as two consecutive PSA elevations with at least 1-week interval, where the PSA applied for judgment is at least 2 ng/mL higher than nadir and total testosterone levels < 50 ng/dL; (2) no history of previous intravenous systemic cytotoxic chemotherapy; (3) ≥20 years when written informed consent is provided; (4) Eastern Cooperative Oncology Group performance status (PS) of 0–2; (5) appropriate renal and hepatic functionality showing serum creatinine ≤ 2.0 × upper limit of normal (ULN), total bilirubin level ≤ 1.5 × ULN, aspartate transaminase ≤ 2.5 × ULN (≤5.0 × ULN in patients with liver metastasis), and alanine transaminase ≤ 2.5 × ULN (≤5.0 × ULN in patients with liver metastasis), and neither ascites nor hepatic encephalopathy are present as demonstrated within 4 weeks before registration, and (6) >3 months life expectancy. Patient ineligibility criteria were (1) desire to have children, (2) a potential allergic reaction to ENZ or ABI treatment, and (3) any other reasons to be inappropriate for participation in the present study judged by a principal or clinical investigator (e.g., cognitive dysfunction). A history of any other treatments was permitted except for cytotoxic intravenous chemotherapies.

Study treatments were terminated when (1) the patient died, (2) PSA progression was confirmed, or (3) AEs occurred. Throughout the study, luteinizing hormone-releasing hormone agonists or antagonists were continued. Denosumab and zoledronic acid were allowed for bone metastatic patients. Sequential treatments were allowed after PSA progression in both arms. Dose reduction was allowed if a principal or clinical investigator judged the standard dose to be inappropriate for any reason (e.g., low body weight).

### 2.3. Definition of Endpoints

The time to PSA progression (TTPP) was defined according to the prostate cancer working group 2 (PCWG2) criteria [5]. Briefly, the PSA progression date was defined as the date when an absolute increase of ≥2 ng/mL and a ≥25% increase above the nadir was documented in patients with PSA levels that declined at week 13. This PSA elevation was confirmed by a subsequent value obtained after at least 3 weeks [5,6]. For patients without a PSA decrease at week 13, the PSA progression date was defined as the date when an absolute increase of ≥2 ng/mL and a ≥25% increase above baseline were documented [5,6]. This was confirmed by a subsequent value after at least 3 weeks. However, in patients with PSA levels that did not decrease, the PSA progression date was defined as the date when the study treatment was discontinued before week 13. TTPP was defined as the time from the randomization date to the first confirmed PSA progression date in all patients. The other endpoints defined as follows were also investigated (1) PSA response rate, defined by ≥50% decline in PSA value from baseline); (2) OS, defined as the time from the randomization date to death from any cause; (3) rPFS according to the Response Evaluation Criteria in Solid Tumors (RECIST), version 1.1, criteria for soft-tissue lesions examined on magnetic resonance imaging or computed tomography scan and using the PCWG2 criteria for bone metastasis examined on bone scintigraphy (these modalities were also used for checking metastasis before the randomization); (4) docetaxel treatment-free survival (DFS), defined as the time from the randomization date to commencement of docetaxel; (5) prostate cancer-specific survival (PCSS), defined as the time from the randomization date to death from prostate cancer; (6) performance status progression-free survival (PSPFS), defined as the time from randomization date to first confirmed PS progression date; and (7) AEs according to the frequency and grade using the Common Terminology Criteria for Adverse Events (CTCAE), version 4.0 http://evs.nci.nih.gov/ftp1/CTCAE/About.html (accessed on 22 January 2024).

### 2.4. Statistical Analyses

A previous report described detailed information about statistical analyses [14]. The Kaplan–Meier method was used to estimate the survival curves. Differences in survival curves between the two patient arms were assessed using a log-rank test. The Cox proportional hazards model was used to estimate the hazard ratio. Fisher’s exact test was used to compare the PSA response rate and the incident proportion of ≥grade 3 AEs between the arms. All tests were two-sided, and a *p* value of 0.05 was considered statistically significant.

## 3. Results

This study enrolled 203 patients from 20 February 2015 to 31 July 2019, and 188 patients were randomly assigned to the ENZ or ABI arm (94 in each arm). The ENZ and ABI arms included 15 and 26 patients with nonmetastatic CRPC, respectively. The data at the cutoff date (22 April 2020) were analyzed, and the median follow-up time was 22.8 months. At the cutoff date, four and eight patients with nonmetastatic CRPC in the ENZ and ABI arms, respectively, died. Table 1 shows the baseline characteristics at randomization. Although the baseline characteristics in both arms were basically well-balanced, the ENZ arm showed a low PSA compared to the ABI arm (median 4.7 ng/mL vs. 7.5 ng/mL), and the ABI arm showed a short duration from castration resistance to randomization compared to the ENZ arm (0.9 months vs. 2.1 months). In addition, the percentage of regional lymph node metastasis was 20% and 38% in the ENZ and ABI arms, respectively. However, no statistical differences were found in the baseline factors between the arms.

**Figure 1:**
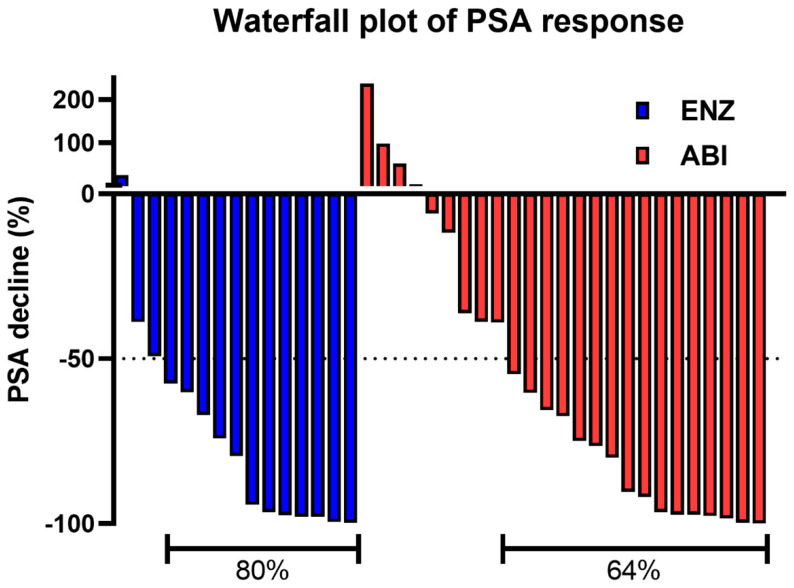
The waterfall plot of the PSA response is defined as a ≥50% decline in the PSA level from baseline. PSA prostate-specific antigen.

TTPP, which is the primary endpoint of the ENABLE Study for PCa, has been already reported. Briefly, the median TTPPs were 33.5 and 27.4 months in the ENZ and ABI arms, respectively. The percentage of patients without PSA progression at 24 months was 59.7% and 55.0% in the ENZ and ABI arms, respectively. No significant difference was observed in TTPP between the two arms (hazard ratio [HR]: 0.56; 95% confidence interval [CI]: 0.21–1.50; *p* = 0.2196) [12]. The PSA response rate, defined as a ≥50% decline in the PSA level from baseline, was analyzed. The results showed that the PSA response rates of the ENZ and ABI arms were 80% and 64%, respectively (*p* = 0.3048; Figure 1).

**Figure 2:**
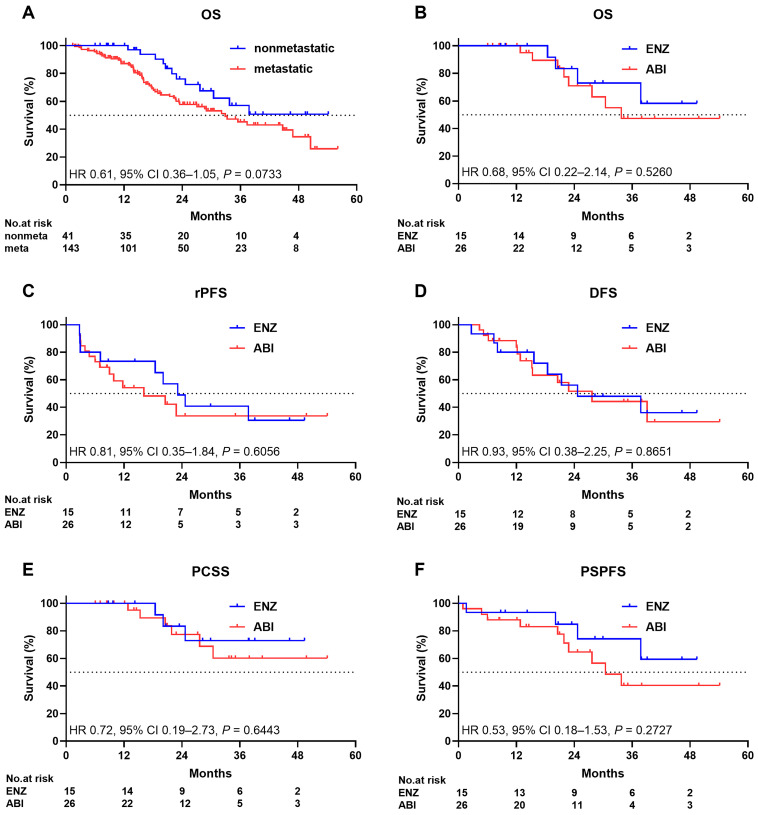
Kaplan–Meier estimate of (**A**) overall survival of patients with nonmetastatic and metastatic disease ((**B**–**F**), ENZ and ABI survival in patients with nonmetastatic disease), (**B**) overall survival, (**C**) radiographic progression-free survival, (**D**) docetaxel-free survival, (**E**) prostate cancer-specific survival, and (**F**) performance status progression-free survival. ENZ enzalutamide, ABI abiraterone plus prednisolone.

The median OS of 41 patients with nonmetastatic CRPC was not reached. The median OS of 143 patients with metastatic CRPC was 32.9 months. The 24-month survival rates were 76.0% and 57.8% in patients with nonmetastatic CRPC and those with metastatic CRPC, respectively. Patients with metastatic CRPC were more likely to have shorter survival than those with nonmetastatic CRPC. However, there was no statistically significant difference in terms of OS between the two groups (HR: 0.61; 95% CI: 0.36–1.05, *p* = 0.0733; Figure 2A).

The median OS of the ENZ arm was not reached, and the median OS of the ABI arm was 33.7 months. The 24-month survival rates were 83.3% and 70.9% in the ENZ and ABI arms, respectively. No significant difference in terms of OS was observed between the two arms (HR: 0.68; 95% CI: 0.22–2.14, *p* = 0.5260; Figure 2B). The median rPFSs were 23.1 and 16.1 months in the ENZ and ABI arms, respectively. Approximately 48.9% and 33.7% of the patients in the ENZ and ABI arms, respectively, did not present with radiographic progression at 24 months. No significant difference in terms of rPFS was observed between the two arms (HR: 0.81; 95% CI: 0.35–1.84; *p* = 0.6056; Figure 2C). DFS and PSPFS are important for assessing the effect of treatment on quality of life in patients, and PCSS is also important for assessing the effect of treatment directly on survival without considering treatment-associated indirect death or death from other comorbidities. The median DFS rates were 24.7 and 27.7 months in the ENZ and ABI arms, respectively. Approximately 56.0% and 51.5% of the patients in the ENZ and ABI arms, respectively, did not receive docetaxel treatment at 24 months. There was no significant difference in DFS between the two arms (HR: 0.93; 95% CI: 0.38–2.25; *p* = 0.8651; Figure 2D). The median PCSS was not reached in either arm. Approximately 83.3% and 77.4% of the patients in the ENZ and ABI arms, respectively, did not receive docetaxel treatment at 24 months. The PCSS did not significantly differ between the two arms (HR: 0.72; 95% CI: 0.19–2.73; *p* = 0.6443; Figure 2E). The median PSPFS was not reached in the ENZ arm, and the median PSPFS of the ABI arm was 30.5 months. Approximately 84.8% and 64.6% of the patients in the ENZ and ABI arms, respectively, did not present with PS progression at 24 months. There was no significant difference in terms of PSPFS between the two arms (HR: 0.53; 95% CI: 0.18–1.53; *p* = 0.2727; Figure 2F).

Four and six patients in the ENZ and ABI arms, respectively, developed ≥grade 3 AEs. The ≥grade 3 AEs in the ENZ arm were anemia, fracture, rupture of the aortic aneurysm, and arrhythmia. The ≥grade 3 AEs in the ABI arm were high aspartate aminotransferase and alanine aminotransferase levels, hypertension, gastric cancer, acute myocardial infarction, and hypokalemia. There were no common ≥grade 3 AEs in either arm. Although malaise and digestive symptoms were frequently observed in the ENZ arm, they were not considered to be severe. Elevated liver enzyme levels or electrolyte imbalance in the ABI arm were also not life-threatening (Table 2).

Approximately 54% and 65% of the patients in the ENZ and ABI arms, respectively, received systemic post-treatment for prostate cancer after study treatment. Docetaxel, which is the second-line treatment, was most frequently used (20%), followed by ABI or radium-223 (13%) in the ENZ arm. ENZ was most commonly administered (27%), followed by docetaxel (23%), in the ABI arm. Subsequent treatments, up to the fifth line, for prostate cancer (including the rechallenge of study treatments) have been reported (Table 3).

## 4. Discussion

This investigator-initiated, multicenter, randomized controlled trial showed no significant differences in not only TTPP but also OS, rPFS, and DFS between the ENZ and ABI arms according to the intention-to-treat (ITT) analysis. However, the ENZ arm had a significantly better PSA response rate than the ABI arm, in addition to the relatively low incidence of severe AEs. Both ENZ and ABI have been used for not only metastatic CRPC but also nonmetastatic CRPC since their approval by the Japanese health insurance system. Therefore, the ENABLE Study could include patients with nonmetastatic CRPC. Importantly, the use of ENZ, apalutamide, and darolutamide was approved after performing an RCT on patients with nonmetastatic CRPC. However, thus far, there is no clinical evidence regarding the use of ABI in patients with nonmetastatic CRPC. ABI is not approved in countries globally, except in Japan [15,16,17].

There was no significant difference in terms of the PSA response rate between the ENZ and ABI arms (80% and 64%, respectively; *p* = 0.30) in patients with nonmetastatic CRPC. Furthermore, there were no significant differences in terms of survival endpoints such as OS, rPFS, DFS, PCSS, and PSPFS between the two arms. As shown in Table 3, systemic post-treatment after study treatments could be regarded as almost similar between both arms when taking a look up to the fifth line. Docetaxel is thought to be a key agent after ARSTs before chemotherapy, and the percentage of docetaxel treatment in second-line and total lines was 20% and 33% in the ENZ arm and 23% and 27% in the ABI arm, respectively [18,19,20]. On the other hand, taxanes and ARSTs have the potential to induce more malignant properties, resulting in visceral metastasis and neuroendocrine differentiation [21,22,23,24,25]. The unique feature of systemic post-treatment after the study treatments in the ABI arm involved more frequent use of dexamethasone and ethinylestradiol. These drugs were reported to improve some outcomes in prostate cancer patients [26,27,28,29]. Dexamethasone was used for 0 and 3 patients in the ENZ and ABI arms, respectively, and ethinylestradiol was used for 2 and 5 patients in the ENZ and ABI arms, respectively. The reason for these deviations in dexamethasone and ethinylestradiol usage is not clear; however, the necessity of prednisolone use in the ABI arm may facilitate changes in steroidal agents. Previous research has shown that metastasis-free survival did not differ across ENZ, apalutamide, and darolutamide, based on indirect comparisons. However, similar to apalutamide and darolutamide, ABI could have a similar survival benefit in nonmetastatic CRPC [30]. Only four and six patients in the ENZ and ABI groups, respectively, presented with ≥grade 3 AEs. The incidence rates of ≥grade 3 AEs did not exceed the expected rate and were acceptable because the RCTs of ARSTs for nonmetastatic CRPC had similar incidence rates for ≥grade 3 AEs [15,16,17]. Meanwhile, the AE profile differed across ARSTs. Apalutamide use was associated with higher rates of falls, fractures, and rashes. Moreover, ENZ, unlike darolutamide, had higher rates of falls, dizziness, mental impairment, fatigue, and severe fatigue [30]. In the current study, malaise, decreased appetite, nausea, and vomiting (although not severe) were frequently observed in the ENZ arm. However, they were also observed in the ENZ arm via ITT analysis. Although the ABI arm showed specific features of AEs, such as a high incidence of elevated liver enzyme levels (aspartate aminotransferase and alanine aminotransferase), hypertension, and rash based on the ITT analysis we reported previously, nonmetastatic CRPC patients in the ABI arm did not show such specific features of AEs. The difference in general status between patients with metastatic CRPC and those with nonmetastatic CRPC in the ABI arm might have contributed to the better AE profile in patients with nonmetastatic CRPC. However, this is an important finding which supports the applicability of ABI to nonmetastatic CRPC. ABI and other ARSTs can be considered as treatments for nonmetastatic CRPC according to the characteristics of the patients or the presence of comorbidities, which could affect the development of AEs. However, this discrepancy should be investigated in further studies. Moreover, direct comparisons between ABI and darolutamide and between ABI and apalutamide should also be performed to better understand the role of ABI.

The current study had some limitations. It only included Japanese patients, and the number of patients was small. In addition, patients with other malignancies were also included. Moreover, since the current study is an open-label study in a real-world clinical setting, potentially biased risks may arise from dose reduction/discontinuation, interpretation of acquired data on the treatment courses, and inappropriate follow-up discontinuation. In addition, the current study included all comorbidities, except for some predefined states, to reflect the real-world nature. The inconsistent methods of each institutional PSA assay might cause biased risks [31]. Attention should be paid to a wide range of PSA values with no statistical difference between arms because the PSA value at baseline itself is a predictor of advanced prostate cancer [32]. These factors reduce the evidential power of survival and safety analyses to some extent.

## 5. Conclusions

The ENABLE Study for PCa first compared the efficacy and safety of ENZ and ABI in patients with nonmetastatic CRPC. The results showed that ABI and ENZ had similar efficacy and safety. This notion supports the applicability of ABI in nonmetastatic CRPC. Nevertheless, further investigations should be performed to obtain actual evidence.

## Figures and Tables

**Table 1:** Baseline characteristics at randomization.

	Variable	ENZ (*n* = 15)	ABI (*n* = 26)
Age (years)		78.3 (66.4–87.0)	77.4 (63.2–92.5)
Performance status			
	0	11 (73%)	19 (73%)
	1	4 (27%)	7 (27%)
Gleason score			
	5, 6	2 (13%)	0 (0%)
	7	2 (13%)	6 (23%)
	8	3 (20%)	2 (8%)
	9	6 (40%)	12 (46%)
	10	2 (13%)	4 (15%)
	Not available	0 (0%)	2 (8%)
Local treatment *			
	Prostatectomy	5 (36%)	3 (12%)
	Irradiation ^†^	2 (14%)	10 (40%)
	None	7 (50%)	12 (48%)
Regional lymph node metastasis			
	Yes	3 (20%)	10 (38%)
	No	12 (80%)	16 (62%)
No. of previous systemic therapy ^‡^		3.0 (2–4)	3.0 (1–4)
Prostate-specific antigen (ng/mL)			
	at diagnosis ^§^	49.9 (16.4–398)	53.8 (5.3–973)
	at nadir before registration ^||^	0.162 (0.003–16.3)	0.143 (0.001–13.8)
	at registration	4.7 (2.1–63.9)	7.5 (2.3–24.0)
Time from diagnosis of prostate cancer to randomization (months) ^¶^		62.0 (14.8–158)	54.6 (9.2–190)
Time from castration resistance to randomization (months) **		2.1 (0.0–99.1)	0.9 (0.0–37.4)

Data are presented as median (range) and *n* (%). Abbreviations: ENZ, enzalutamide; ABI, abiraterone plus prednisolone. * Data of 1 in ABI are not available. ^†^ Including high and low dose rate brachytherapy and external beam radiation therapy for the primary site. ^‡^ Medical or surgical castration is counted as 1, and data of 1 in ABI is not available. ^§^ Data of 1 in ABI are not available. ^||^ Data for 1 in the ENZ and 2 in the ABI are not available. ^¶^ Data of 2 in the ENZ and 1 in the ABI group are not available. ** Data of 4 in ENZ and 3 in ABI are not available.

**Table 2:** All adverse events.

	ENZ (*n* = 15)		ABI (*n* = 26)	
Event	Any Grade	Grade ≧ 3	Any Grade	Grade ≧ 3
Anemia	3 (20%)	1 (7%)	4 (15%)	0
Thrombocytopenia	1 (7%)	0	0	0
Malaise	4 (27%)	0	2 (8%)	0
Fatigue	1 (7%)	0	2 (8%)	0
Decreased appetite	3 (20%)	0	1 (4%)	0
Nausea	2 (13%)	0	0	0
Vomiting	1 (7%)	0	0	0
Body weight loss	1 (7%)	0	2 (8%)	0
Increased aspartate aminotransferase	1 (7%)	0	1 (4%)	1 (4%)
Increased alanine aminotransferase	0	0	1 (4%)	1 (4%)
Fracture	1 (7%)	1 (7%)	1 (4%)	0
Hypertension	1 (7%)	0	1 (4%)	1 (4%)
Edema	0	0	2 (8%)	0
Diarrhea	1 (7%)	0	0	0
Constipation	1 (7%)	0	1 (4%)	0
Hot flash	0	0	1 (4%)	0
Pruritus	1 (7%)	0	0	0
Gastric cancer	0	0	1 (4%)	1 (4%)
Rupture of aortic aneurysm	1 (7%)	1 (7%)	0	0
Acute myocardial infarction	0	0	1 (4%)	1 (4%)
Arrhythmia	1 (7%)	1 (7%)	0	0
Atrial fibrillation	1 (7%)	0	0	0
ST elevation in electrocardiogram	1 (7%)	0	0	0
Renal disorder	1 (7%)	0	0	0
Dehydration	0	0	1 (4%)	1 (4%)
Hypokalemia	0	0	1 (4%)	0
Hyperglycemia	0	0	1 (4%)	0
Numbness	1 (7%)	0	0	0
Myalgia	0	0	2 (8%)	0
Hematuria	0	0	1 (4%)	0
Gallbladder wall thickness	1 (7%)	0	0	0
Sleep disorder	0	0	1 (4%)	0
Dizziness	0	0	1 (4%)	0
Headache	1 (7%)	0	0	0
Seizure	1 (7%)	0	1 (4%)	0

Data are presented as *n* (%). Abbreviations: ENZ, enzalutamide; ABI, abiraterone plus prednisolone.

**Table 3:** Systemic post-treatment for prostate cancer after study treatments.

	Treatment	ENZ (*n* = 15)	ABI (*n* = 26)
Second line			
	(study treatment continued)	5 (33%)	6 (23%)
	None	2 (13%)	3 (12%)
	Abiraterone + prednisolone	2 (13%)	0
	Enzalutamide	0	7 (27%)
	Docetaxel	3 (20%)	6 (23%)
	Ethinylestradiol	0	3 (12%)
	Radium-223	2 (13%)	0
	Apalutamide	1 (7%)	0
	Dexamethasone	0	1 (4%)
Third line			
	Abiraterone + prednisolone	1	1
	Enzalutamide	0	3
	Docetaxel	0	1
	Ethinylestradiol	2	1
	Apalutamide	1	0
	Dexamethasone	0	1
	Cabazitaxel	1	2
Fourth line			
	Abiraterone + prednisolone	1	0
	Docetaxel	2	0
	Ethinylestradiol	0	1
	Dexamethasone	0	1
	Cabazitaxel	0	1
Fifth line			
	Enzalutamide	0	1
	Cabazitaxel	2	0

Abbreviations: ENZ, enzalutamide; ABI, abiraterone plus prednisolone.

## Data Availability

Kouji Izumi had full access to all the data in the study and takes responsibility for the integrity of the data and accuracy of the data analysis.
